# In-Depth Study of the Interaction, Sensitivity, and Gating Modulation by PUFAs on K^+^ Channels; Interaction and New Targets

**DOI:** 10.3389/fphys.2016.00578

**Published:** 2016-11-24

**Authors:** Cristina Moreno, Alicia de la Cruz, Carmen Valenzuela

**Affiliations:** ^1^Department of Cardiology, Cardiovascular Research Institute Maastricht, Maastricht University Medical CentreMaastricht, Netherlands; ^2^Departamento de Modelos Experimentales de Enfermedades Humanas, Instituto de Investigaciones Biomédicas “Alberto Sols” CSIC - Universidad Autónoma de MadridMadrid, Spain

**Keywords:** PUFAs, shaker K channels, Kv7.1, K2P, KCA, KATP, lipoelectric hypothesis

## Abstract

Voltage gated potassium channels (K_V_) are membrane proteins that allow selective flow of K^+^ ions in a voltage-dependent manner. These channels play an important role in several excitable cells as neurons, cardiomyocytes, and vascular smooth muscle. Over the last 20 years, it has been shown that omega-3 polyunsaturated fatty acids (PUFAs) enhance or decrease the activity of several cardiac K_V_ channels. PUFAs-dependent modulation of potassium ion channels has been reported to be cardioprotective. However, the precise cellular mechanism underlying the cardiovascular benefits remained unclear in part because new PUFAs targets and signaling pathways continue being discovered. In this review, we will focus on recent data available concerning the following aspects of the K_V_ channel modulation by PUFAs: (i) the exact residues involved in PUFAs-K_V_ channels interaction; (ii) the structural PUFAs determinants important for their effects on K_V_ channels; (iii) the mechanism of the gating modulation of K_V_ channels and, finally, (iv) the PUFAs modulation of a few new targets present in smooth muscle cells (SMC), K_Ca_1.1, K_2P_, and K_ATP_ channels, involved in vascular relaxation.

## Introduction

**Figure 1:**
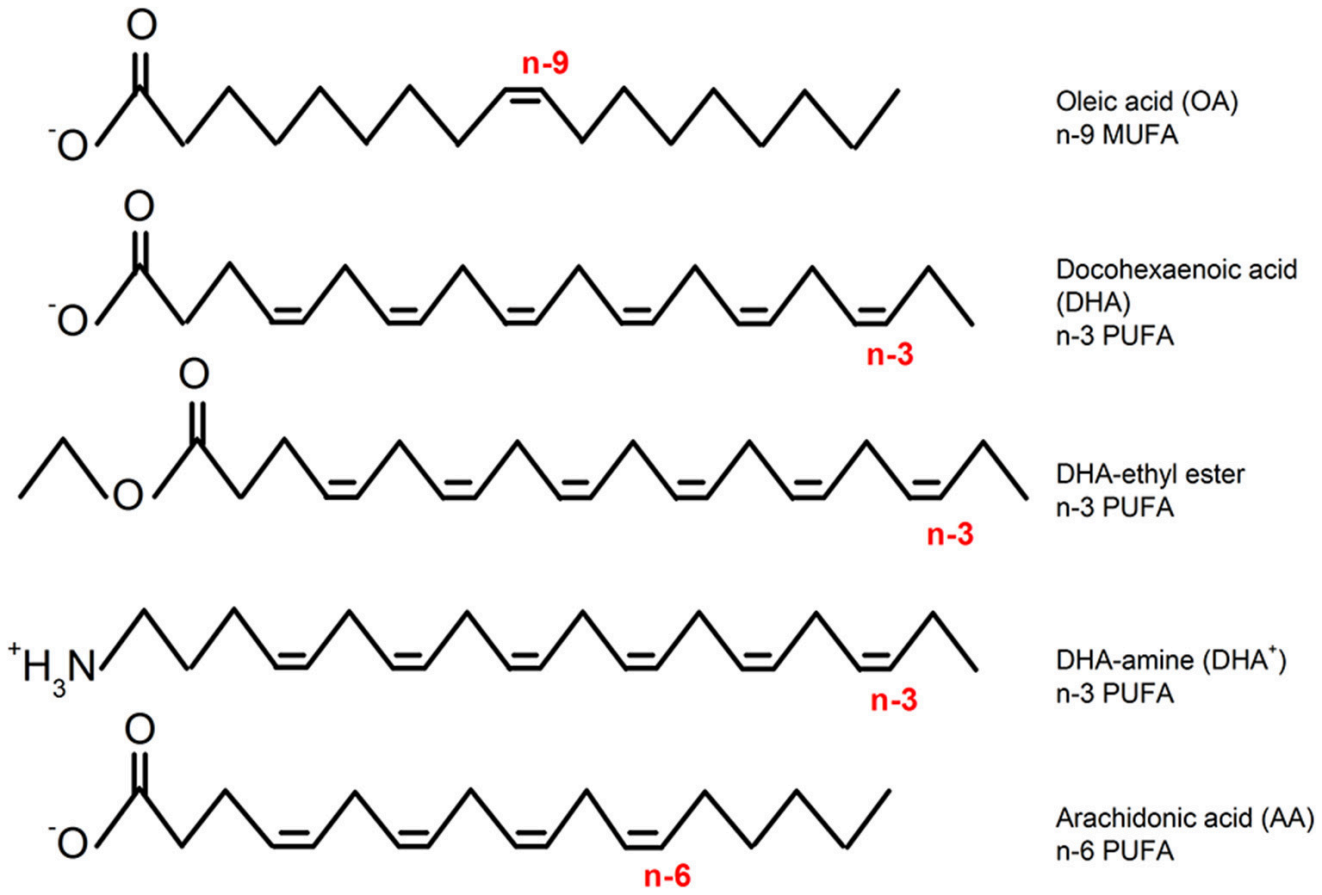
**Chemical structures of representative fatty acids (monounsaturated: n-9 MUFA, n-3, and n-6 PUFAs) and modified PUFAs (DHA-ethyl-ester DHA and DHA-amine: non-charged and permanently charged, respectively)**.

It has been reported that an increased consumption of omega 3 polyunsaturated fatty acids (n-3 PUFAs; Figure 1) have beneficial properties for the cardiovascular system (Chaddha and Eagle, 2015) and neurological diseases such as epilepsy and pain (Lefevre and Aronson, 2000). Among other targets, the beneficial actions of n-3 PUFAs occur through the modulation of a big variety of K^+^ voltage gated ion channels (Boland and Drzewiecki, 2008; Moreno et al., 2012). For instance, it is known that n-3 PUFAs activate different members of the K_V_7 channel family. In the heart, n-3 PUFA dependent activation of K_V_7.1 channels (pore forming component of the cardiac *I*_Ks_) reduces the risk of arrhythmia by shortening the action potential duration (Verkerk et al., 2006; Liin et al., 2015; Moreno et al., 2015). In the neuronal system, the activation of K_V_7.2/K_V_7.3 channels (major components of the neuronal M-current) decreases neuronal excitability and therefore the risk of seizure (Liin et al., 2016; Valenzuela, 2016).

Despite the encouraging results of the first clinical trials published in the early 2000s about n-3 PUFAs protective effects, very soon other studies reported no beneficial or even harmful effects. For example, an increased risk of cardiac events was found in angina pectoris patients (Burr et al., 2003). The physiological effects of PUFAs are complex and poorly understood. In the context of excitable diseases, we believe that the conflicting results emerged from the lack of exhaustive knowledge on two aspects of the n-3 PUFA-dependent modulation of voltage-gated ion channels. The mechanisms by which n-3 PUFAs differentially modulate ion channels and for which specific pathologies n-3 PUFAs are truly beneficial are poorly understood. Different cardiovascular and neuronal pathologies are triggered by different underlying mechanism. Therefore, a diet rich in n-3 PUFAs might be beneficial for some conditions but neutral or deleterious for others.

The mechanism of action by which PUFAs modify ion channel activity has been debated for years. Several theories, ranging from (1) non-specific effects on the cell membrane (Leifert et al., 1999) to (2) specific binding to the ion channel (Hallaq et al., 1992; Kang and Leaf, 1996; Bendahhou et al., 1997; Xiao et al., 2005; Guizy et al., 2008), have been proposed. Scientific evidence in favor of the indirect modulation of ion channels by n-3 PUFAs are the lack of selectivity in blocking a given channel *vs*. other, and the reported effects on the cell membrane fluidity. On the other hand, the identification of residues in the ion channel protein involved in n-3 PUFAs sensitivity argues in favor of the second theory. A great step forward defining the molecular mechanism of n-3 PUFAs modulation of K_V_ channels has been recently achieved. The identification of the PUFA binding site on several K^+^ channels, the proposed mechanism of action for the direct effects of PUFAs and the experimental evidence showing that n-3 PUFAs can modify the gating of ion channels simultaneously by establishing direct interactions and by modifying the properties of the lipid bilayer were essential findings that helped to clear up the controversy (Borjesson et al., 2008; Moreno et al., 2015).

Important advances have been achieved in the identification of the specific pathologies that could benefit from a diet rich on n-3 PUFAs as well. In this regard, many new ion channel targets in different organs and tissues have been identified, and disease animal models have been produced to understand how PUFAs can affect physiology.

In the present review we first summarize results identifying the PUFA binding site on K^+^ ion channels, the molecular determinants that determine the affinity and potency within the n-3 PUFA molecule, and the proposed mechanism of action by which n-3 PUFAs modify the gating K^+^ channels: the “lipoelectric hypothesis.” In the second part of the review, we focus on recent findings in PUFAs-dependent modulation of the K_V_7 family and a few new targets: K_2P_, K_Ca_1.1, and K_ATP_ in the cardiovascular and neuronal function. Finally, we briefly discuss how the PUFAs represent a new pharmacological treatment in which ion channel voltage dependence is electrically modulated instead of the more traditional pore block.

## Selective PUFA effects on different potassium channels

In this section we recapitulate the arguments in favor of specific PUFA-ion channel interactions emphasizing findings published in last 5 years. We will focus on two potassium channels *Shaker* and K_Ca_1.1, for which the PUFA induced modulation of ion channels, has been more extensively characterized.

### Evidences for selective PUFAs effects on *Shaker* K^+^ channels

After the initial studies based on the introduction of point mutations in the ion channel to demonstrate point direct interaction between the ion channel and the PUFA (Xiao et al., 2001a,b), another solid evidence showing how PUFAs differently modify ion channels raised from the observation that the potency of the n-3 PUFA docosahexaenoic acid (DHA) on *Shaker* voltage dependence changes with local pH (Borjesson et al., 2008). At physiological pH, DHA increased *Shaker* K^+^ current by shifting the midpoint of activation [G(V) curve] toward hyperpolarized potentials. It is known that different ion channels have different local pH values depending on their local set of surface charges structure (Elinder et al., 1996). To explore the influence of the surface charges and local pH on *Shaker* modulation by DHA, Borjesson and colleagues used a mutated *Shaker* channel where residues A419, F425, and V451 were made positive (Borjesson et al., 2008). The presence of these three positive residues in the extracellular loops connecting transmembrane segments S5 and S6 of *Shaker* K^+^ channels resulted in a local pH change of 0.3. Under these conditions, DHA induced a negative shift of the midpoint of activation in the triple mutant about twice as large as the shift induced in the WT *Shaker* K^+^ channel. These results clearly indicate that the PUFA-induced effect is channel specific and depends on the channel-specific set of surface charges (Borjesson et al., 2008).

**Figure 2:**
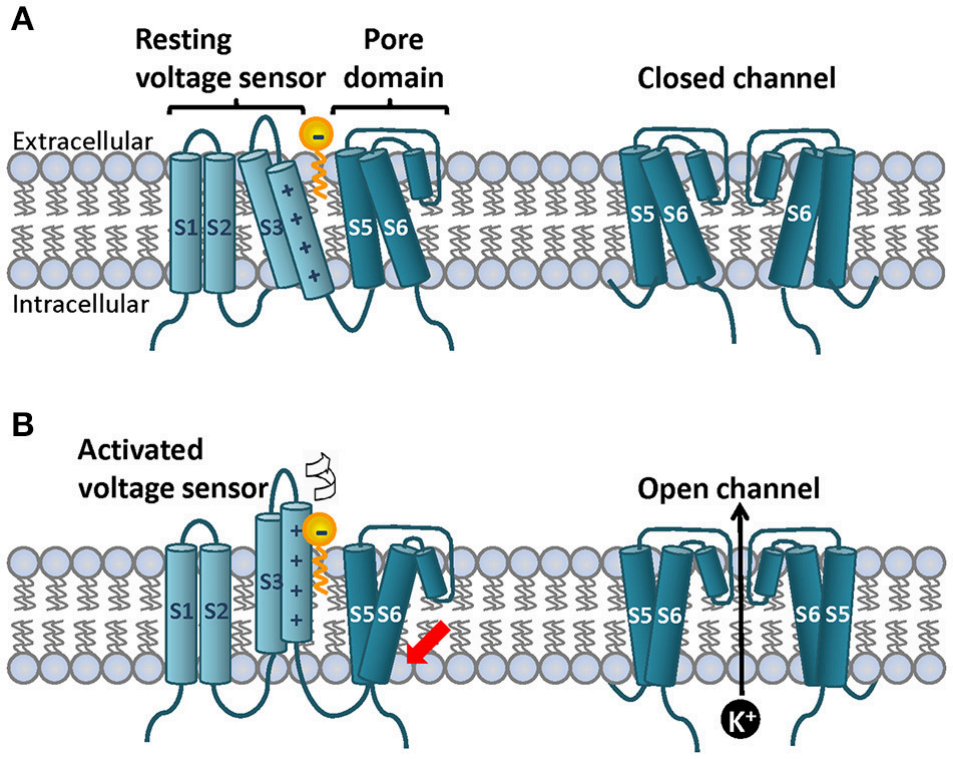
**Topology of a canonical voltage gated ion channel subunit on the lipid bilayer and cartoon representation of the lipoelectric hypothesis mechanism of PUFA-dependent modulation of ***Shaker***-like K^**+**^ channels**. On the cell membrane four subunits co-assemble to for the ion channel. **(A)** Each subunit contains six transmembrane segments and N-and C-terminal domains. S1–S4 segments form the voltage sensor and S5–S6 form the ion pore. The S4 segment contains a variable number of positively charged residues known as gating charges that detect small changes on the electric field on the membrane. In response to prolong depolarizations the S4 moves upward perpendicular to the membrane and rotates. **(B)** This process is called voltage sensor domain activation and it is indicated by the white arrow. Once the four individual S4 are on the upstate the activation gate located at the base intracellular site of the pore domain can open to allow K^+^ flow (red arrow). A simplified PUFA structure is depicted with the head group located at the extracellular bilayer interface and the bilayer center is depicted in yellow. The PUFA head group interacts electrostatically with the upper gating charges of the S4 promoting mainly channel opening (red arrow). Top and bottom right panels show the front view of the S5 and S6 segments in the closed and open state. Two subunits are depicted for simplicity.

The ultimate proof for the specific PUFAs effects on different ion channels came with the identification of the PUFAs binding site on *Shaker* channels. Because of its lipophilic character and modulatory effects it was proposed that the PUFAs binding site on *Shaker* K^+^ channels should be in the vicinity of the gating charges in the voltage sensor (S4 segment) and near residues facing the lipid bilayer (Borjesson et al., 2008). The confirmation for the proposed binding site came quickly. A cysteine scan analysis covering the lipophilic surfaces of the extracellular halves of S3–S6 segment showed that residues I325C and T329C located in the carboxyl end of helix S3, and I360C at S4 were insensitive to DHA. On the contrary, the L366C mutation increased DHA sensitivity of *Shaker* K^+^ channels (Borjesson and Elinder, 2011). To further define the PUFAs binding site, positives charges were introduced to each of the above-mentioned residues in the S3–S4 regions by using MTSEA^+^ reagent. Consistently with the cysteine scan data, a positive charge at residues I325; T329 and A359; I360 of the S3 and S4 respectively resulted in an increased sensitivity to DHA effects (Borjesson and Elinder, 2011). In addition to the experimental data, a structural model built to predict 3D interactions suggested that a negative charge at R1 (R362) would reduce the PUFA effect. Consistently with the model, when the charge of R362C mutant was modified negatively with MTES^−^ reagent the G(V) shift induced by DHA was smaller than that induced in WT *Shaker* K^+^ channels. In contrast, R362C^+^ (exposed to MTSET^+^) restored PUFA sensitivity. Some K_V_ channels such as K_V_1.2 have an additional gating charge R0 at the top of the S4 segment. An homology model of the *Shaker* K^+^ channel based on the K_V_1.2/2.1 chimera predicts that a positive residue at that position (A359) could strengthen the interaction between the PUFA head group and the ion channel (Borjesson and Elinder, 2011). When attaching MTSET^+^ to A359C, the DHA-induced G(V) shift was greater. Experiments in which the charge of R0 and R1 was changed support the proposed localization of the PUFA binding site and suggested that different PUFAs should have very different effects in modulating different ion channels depending on the presence of a charge at positions R0 and/or R1 (Borjesson and Elinder, 2011). Together, the data showed that high impact residues for DHA on *Shaker* K^+^ channels are clustered in a small region of the lipid facing S3–S4 corner of the voltage sensor domain but distant from the pore region (Figure 2). Positive charges located close to the PUFAs binding site increase PUFA potency.

As shown for *Shaker* K^+^ channels, it has been reported that PUFAs target the voltage sensor domain of many other K_V_ channels such as K_V_7.1 channels (Liin et al., 2015). In K_V_7.1 channels, PUFAs interact with extracellular positively charged residues of the S4 segment to promote channel opening. Neutralization of the most external gating charge (R1) of the S4 of K_V_7.1 channels, R228Q, made K_V_7.1 channels insensitive to DHA, to DHA-glycine (a permanently deprotonated DHA analog at physiological pH due to its lower pKa value) and to N-arachidonoyl taurine (with a permanent negative charge at pH = 7.4). Besides the S4 gating charge residues, other amino acids located in the outer halves of S3 and S4 had a big impact in the PUFA modulatory effect. Again a cysteine scan analysis showed that residues K218C and G219C, located in the S3–S4 extracellular linker of K_V_7.1 reduced the PUFA effect by a factor of 3, suggesting the existence of PUFA binding site equivalent in other K_V_ channels (Liin et al., 2015).

### Evidences for selective PUFAs effects in hK_Ca_1.1 channels

Although in general different potassium channels show high similarity regarding structural domains and gating mechanisms, some important differences exist between different K_V_ families. One proof of that is the different location of the PUFA binding site on different K_V_ families as demonstrated for hK_Ca_1.1 channels (or large conductance Ca^2+^- activated K^+^ channels).

In a study by Hoshi and colleagues it was shown that DHA increased hK_Ca_1.1 channels by 2.5-fold and induced a shift of the G(V) curve into the negative direction (Hoshi et al., 2013c). Regardless the potentiating effects of DHA on hK_Ca_1.1 channels K_Ca_1.1 channels from Drosophila were insensitive to DHA. In order to determine the molecular determinants within hK_Ca_1.1 channels critical for the stimulatory effect, chimeric constructions of Human/Drosophila hK_Ca_1.1 channels were done. The chimeric constructions revealed that the PUFA binding site is located in the pore domain (S5, P-loop and S6 segments) of hK_Ca_1.1 channels. Furthermore, the introduction of Human-to-Drosophila point mutations in the pore domain of hK_Ca_1.1 highlighted the residue Y318S (located in the ion pore) as a crucial determinant of the differential sensitivity of hK_Ca_1.1 channels compared to dK_Ca_1.1 to PUFAs (Hoshi et al., 2013c).

**Figure 3:**
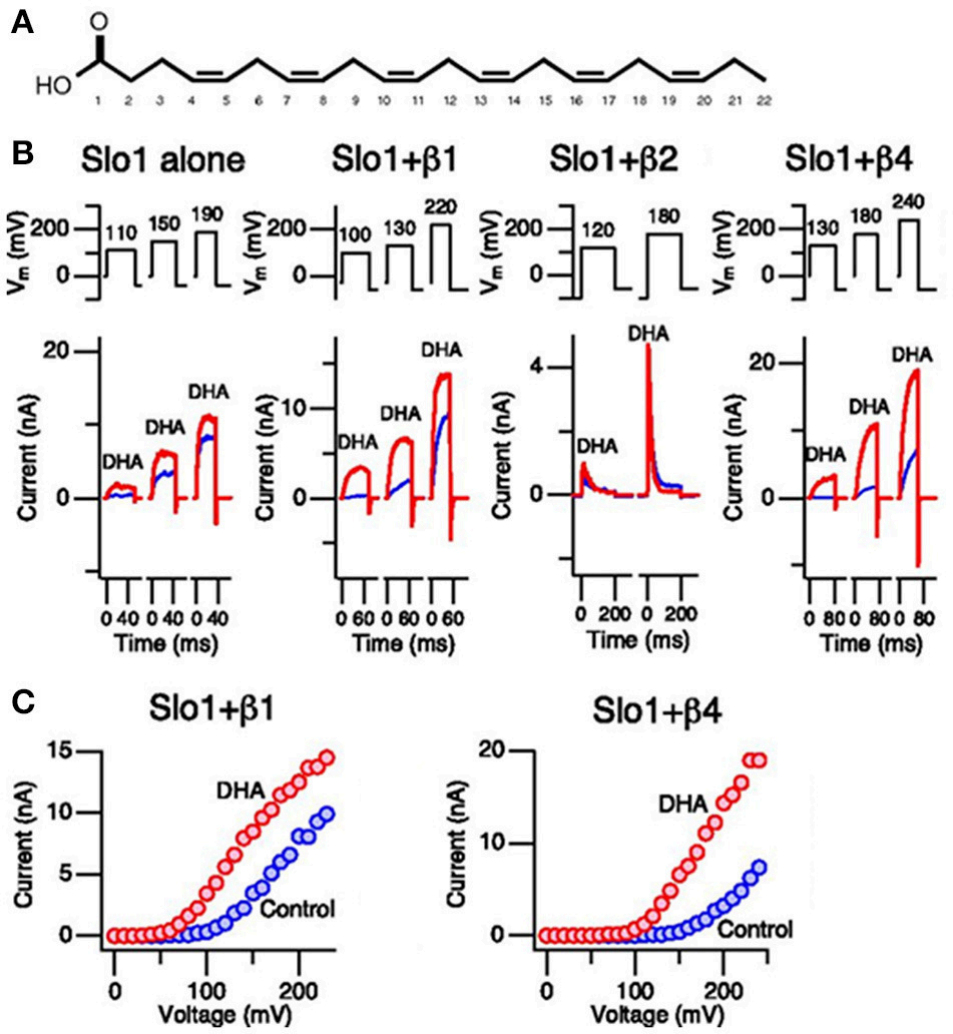
**Effects of DHA on K_**Ca**_1.1 channels. (A)** Chemical structure of DHA. **(B)** Currents generated by K_Ca_1.1, K_Ca_1.1+β1, and K_Ca_1.1+β4 channels in inside-out patches recorded in the absence of calcium. Current records under control conditions and after addition of DHA (3 μM) are shown in blue and red, respectively. **(C)** Current-voltage relationships obtained from K_Ca_1.1+β1 and K_Ca_1.1+β4 are shown. Again, results from control and DHA are shown in blue and red, respectively. Hoshi et al. (2013a)

Besides the identification of the PUFA binding site in K_Ca_1.1 channels, additional evidence in favor of a selective action of PUFAs in hK_Ca_1.1 channels raises from the different potency shown by the fatty acids in hK_Ca_1.1 channels co-expressed with different modulatory subunits. In different tissues, hK_Ca_1.1 channels are co-assembled with β1, β2, β4, or Υ subunits (Wu and Marx, 2010). Another study performed by Hoshi and colleagues, showed a 2.5-fold current density increase induced by DHA on hK_Ca_1.1 channels (expressed in the absence of modulatory subunits), while it increased hK_Ca_1.1+β1 and hK_Ca_1.1+β4 by up to 20-fold and shifted the G(V) by −60 mV. On the contrary, DHA was much less effective in hK_Ca_1.1+β2 and hK_Ca_1.1+Υ1 channels (Figure 3). These differential effects are very noteworthy because all β subunits share a similar amino acid sequence and structural organization. Chimeric experiments determined that the N-terminus and the first transmembrane domain of β1 and β4 subunits are crucial for DHA sensitivity. Finally, point mutations experiments in which amino acids in β1 or β4 were substituted with that in β2 at the corresponding location revealed that R11/E12 and C18/R19 are crucial for DHA sensitivity in β1/β4 respectively. In addition, β2–β1 or β4 substitutions at the equivalent positions were made which showed indistinguishable sensitivity to DHA compared to β1 and β4 subunits (Hoshi et al., 2013a). All these results may suggest that PUFAs binding site resides in both the α and in the β1 and β4 subunits of the hK_Ca_1.1 ion channel complex.

## PUFAs structural determinants important for their effects on ion channels

In parallel to the progression in the understanding of the molecular determinants within the ion channels involved in interactions with PUFAs, several publications have contributed to the identification of the features within the PUFAs molecules important for their effects. The PUFA molecule consists of an aliphatic chain (of variable length) with two or more double bonds capped at the end with a negatively charged carboxyl group. Which are the crucial features for the modulation of K^+^ channels?

### The charge of the carboxyl group is crucial for the electrostatic interaction with the voltage sensing domain of the ion channel

PUFAs such as DHA and arachidonic acid (AA) are negatively charged at physiological pH 7.4. In order to test the effect of the charge of the carboxyl group, Borjesson et al. (2008) compared the effects of DHA (negatively charged), DHA methyl ester (non-charged transesterified DHA), and a charged lipolytic toxin GsMTx4^+^ (structurally similar to PUFAs but with four positive charges) on *Shaker* channels. DHA shifted the G(V) curve by 10 mV toward the negative potentials. DHA methyl ester had no effect on the voltage dependence. Conversely, the GsMTx4^+^ toxin shifted the G(V) curve 5.7 mV into the positive direction. In the same line of evidence it was shown that the potency of DHA changes with pH. A higher pH potentiates DHA effects because a larger fraction of the PUFA is deprotonated (Borjesson et al., 2008). In another study, the pH-dependence of the modulation of *Shaker* by arachidonoyl amine (AA^+^) was analyzed (Borjesson et al., 2010). AA^+^ shifted the G(V) curve of *Shaker* K^+^ channels toward positive potentials and decreased the maximum conductance. AA methyl ester had no effect. Following a similar reasoning, if there is a pH-dependence for the amine effect AA^+^ should be more effective at low pH. As expected, AA^+^ was more potent at a lower pH when a larger fraction of AA^+^ is protonated (Borjesson et al., 2010).

The importance of a negative charge on the head group of DHA is applicable to many other PUFAs but also to many other potassium channels. In a recent study by Liin et al. (2015), it was shown that DHA (negatively charged) shifted the G(V) by ~ −9 mV of K_V_7.1 channels. On the contrary, DHA^+^ and AA^+^ shifted the G(V) by ~ +9 mV. Together, these results demonstrate that the modulation of the voltage dependence of activation of K^+^ channels toward hyperpolarized or depolarized potentials is determined by the valence of the charge in the carboxyl head group. A negative charge shifts the G(V) curve toward more negative potentials and vice versa.

### Two or more cis double bonds in the acyl chain are necessary to shift G(V) curve

Borjesson and colleagues tested ten fatty acids or fatty acid-like compounds with different acyl chain lengths and different numbers, position, and geometry of double bonds to identify structural features on the acyl tail important for the PUFA modulation of *Shaker* K^+^ channels. Fatty acids with the most structural similarity to DHA by having two or more methylene-interrupted double bonds at either the n-3 or the n-6 position showed similar shifts of the G(V) curves in *Shaker* channels (Borjesson et al., 2008). The rest of the compounds were not effective.

Recently, Yazdi et al. (2016) studied how the saturation levels in the carbon tail affect the structure of the lipids in 1-palmitoyl-2-oleoyl-sn-glycero-3-phosphocholine (POPC) membrane patches. From their molecular dynamics simulations the following conclusions were extracted: (i) the order parameters of PUFA and saturated fatty acids decreased from the carboxyl end located in the bilayer interface to the end of the acyl chain tail in the bilayer center. (ii) The order parameters displayed a different overall shape reflecting the positions of the cis double-bonds of PUFAs. (iii) There are differences in the shape and packing properties by measuring the radius of gyration of the carbon tail and head-to-tail length. Compared to other fatty acids, the PUFA molecule tends to twirl and curl up. All together, these results indicate that the PUFA molecule has greater conformational mobility compared to saturated fatty acids. The predominant saturated fatty acid conformation is more extended resulting in a decreased flexibility and mobility of phospholipids of the cell membrane (Yazdi et al., 2016).

### Effect of the n-3 PUFA acyl chain length

Contrary to the importance of a negatively charged carboxyl group for the PUFA effect and the presence of two or more double bonds, the impact of the length of the acyl tail for the PUFAs effects is not so obvious. In *Shaker* K^+^ channels the presence of two or more double bonds and its location in cis geometry (more flexible) seem to be more important than the number of carbons of the tail for the PUFA induced G(V) shift (Borjesson et al., 2008). Similar findings were shown by Liin et al. in K_V_7.1 channels (Liin et al., 2015). Two fatty acids: EPA and oleic acid (OA), poly- and mono-unsaturated fatty acids respectively with similar acyl tail length showed very different effects on K_V_7.1 currents. OA had no effect on K_V_7.1 currents while EPA showed a similar potency increasing K_V_7.1 compared to DHA (Liin et al., 2015). On the other hand, a study by Hoshi et al. (2013c) in which the effects of three n-3 PUFAs: DHA, EPA and α-linolenic acid (ALA) were examined in hK_Ca_1.1 channels revealed that DHA had the largest stimulatory effect on hK_Ca_1.1 currents followed by EPA and finally ALA. These results suggest the importance of the length of the acyl might be channel specific and that the longer the acyl tail, the larger the effect of the PUFA will be.

## Mechanism of gating modulation: lipoelectric hypothesis

### Origin of the lipoelectric hypothesis and definition

Based on the following effects of DHA on *Shaker* K^+^ channels: (i) shift of the G(V) and of the steady state inactivation curve to the negative direction, and (ii) acceleration of the activation kinetics and slower deactivation kinetics; Borjesson and colleagues formulated in 2008 the “Lipoelectric hypothesis” to explain the PUFAs-dependent modulation of K^+^ channels mechanism. All these findings are in agreement with DHA and other PUFAs causing electrostatic effect on the voltage sensor of ion channels (Borjesson et al., 2008). The lipoelectric hypothesis proposes that the lipophilic PUFAs bind to a hydrophobic environment (the lipid bilayer or bilayer/channel interface close to the S3-S4 segment) from where they act electrostatically on the voltage sensor changing the voltage dependence of the ion channel (Borjesson et al., 2008).

### Which activation steps are affected by PUFAs?

It is known that the activation of *Shaker* K^+^ channels occur in at least two steps. In the early transitions the four *Shaker* subunits go through transitions between the closed states (C0 to C2) and, then the channel goes through other two transitions to the final closed and the open states (C2 to O). The transitions between the first closed states are sequential and represent the activation of the voltage sensor, whereas those between C2 to O are concerted and couple the movement of the main voltage sensors to channel opening (Schoppa et al., 1992; Bezanilla et al., 1994; Zagotta et al., 1994; Schoppa and Sigworth, 1998).

$$\begin{array}{l} \;\alpha_{0}\;\;\;\;\alpha_{1}\;\alpha_{1}\text{ ​ }\alpha_{1}\;\alpha_{2}\;\alpha_{3}\;\alpha_{4}\\ \text{C}0↔\text{C}1↔\text{C}11↔\text{C}12↔\text{C}2↔\text{C}3↔\text{C}4↔\text{O}\\ \;\beta_{0}\;\beta_{1}\;\beta_{1}\;\beta_{1}\;\beta_{2}\;\beta_{3}\;\beta_{4} \end{array}$$

To explore which activation step is affected by DHA, gating currents were measured in two mutated *Shaker* K^+^ channel constructions. The final transitions (from close C2 to open O) were studied in the ILT mutant *Shaker* channel where three hydrophobic residues in S4 are substituted for three other hydrophobic residues (V369I, I372L, and S376T). In the ILT mutant the voltage sensor activation and pore domain opening steps are energetically separated allowing the study of the late transitions during the channel activation in isolation The initial steps of activation (C0 to C2) were studied in the non-conducting channel resulting from the ILT mutant plus the pore mutation W343F. DHA significantly shifted the G(V) curve in the negative direction along the voltage axis. On the contrary, DHA had a much smaller effect on the gating currents Q(V) of the ILT/W343F *Shaker* mutant. The larger effect of DHA seen on the G(V) compared to the Q(V) curve suggests that the opening step (the second gating component) is more affected than the early transition by the PUFA (Borjesson et al., 2008). Despite DHA had a much smaller effect on the gating currents, this small effect indicates that the activation of independent voltage sensors is also affected. A gating model of *Shaker* K^+^ channels predicted a DHA-dependent shift of 30 and 5 mV in the G(V) and Q(V) respectively corroborating the experimental data (Borjesson and Elinder, 2011).

### PUFA head group is closer to *Shaker* K^+^ channel in the open state

PUFAs interaction sites in the open and closed state were determined by steered molecular dynamics simulations of *Shaker* K^+^ channels in POPC bilayers (Yazdi et al., 2016). PUFAs form clusters around the voltage sensor domain regions (VSD) rather than the pore helices S5–S6. In general, significantly higher contact frequencies were identified between the PUFA and the protein in the open state. PUFA acyl tail-ion channel protein contacts are predominantly non-specific: hydrophobic and distributed across the VSD. PUFA heads-protein interactions are more frequent in the open *vs*. closed state and are more specific: hydrogen bonds between charged or polar residues of the extracellular halves of S3 and S4 and PUFA head groups. Saturated fatty acids established significantly fewer interactions in closed *vs*. open state simulations and fewer contacts in the open state compared to PUFAs. In summary, specific protein residues involved in PUFAs *vs*. saturated fatty acids interactions are largely different. While PUFAs establish predominantly contacts with S3–S4 residues, there is a shift to the S1–S2 in saturated fatty acids (Yazdi et al., 2016).

### Place dependence and rotation of the first gating charge R1 for the PUFA effect

According to the lipoelectric hypothesis, the PUFA molecule act electrostatically on positive residues of the voltage sensor domain among which arginine 1 (R1) seems to have a crucial role. Shifting the R1 from position 362–361 eliminated the enhancing DHA effect. Moving the charge 2 steps further potentiated the PUFA effect (Ottosson et al., 2014). In the same line of evidence it was found that the introduction of a negative charge (substitution to glutamic acid) in certain positions of the S4 such as A359E, had an opposite effects on the G(V) curve (smaller hyperpolarizing shift compared to WT *Shaker* K^+^ channels) to the potentiated effect of arginine (Ottosson et al., 2014). Even more interestingly, the application of AA^+^ that was previously shown to decrease WT *Shaker* K^+^ current now it up-regulated A359E channels and shifted the G(V) curve −8 mV strongly indicating an electrostatic interaction. AA^+^ did not up-regulated A361E showing again amino acid side facing dependence. A positive charge at 359 or a negative at 361 promotes channel opening and vice versa (Ottosson et al., 2014).

According to the structural prediction by Borjesson and colleagues, *Shaker* K^+^ channels, with all S4s in an activated but non-conducting position, R1 and R2 are exposed to the extracellular solution with its charges at a distance of 16 Å away from the approximate PUFA position in the lipid bilayer adjacent to S3 and S4 (Borjesson and Elinder, 2011). Assuming that the final position of S4 is according to the K_V_1.2/2.1 chimera crystal structure (Long et al., 2007), the charge of R1 moves to a position only 6 Å away from the PUFA when the channel is open. A modified Coulomb law was used to estimate the distance between the PUFA carboxyl charge and the S4 mediated change in surface as a function of distance from a charge. The predicted 5 mV effect on the first four independent transition steps suggests that the PUFA charge is located 15.2 Å away from the position where the positive charges emerge on the channel protein's surface. This calculation was based on the assumption that the channel surface is smooth and that the top S4 charges turn up on the surface of the channel leaving the inner part of the ion pore with positive gating charges pairing with negative counter charges. This is very close to the structural prediction of 16 Å. The 30 mV effect on the opening step suggests that the PUFA charge is located 6.3 Å away from R1 in the open state, which again is almost identical to their prediction from the structural model.

Collectively the data strongly suggest that the late transitions (from C2 to O) are the more sensitive steps to DHA. In the last closed state (C4), R1 and R2 are exposed to the extracellular solution 16 Å away from the PUFA head group. In the opening step, R1 moves along the longitudinal axis of S4 and rotate toward the lipid bilayer, in the vicinity of the negatively charged bound PUFA (6 Å; Figure 2). DHA electrostatically affects this rotation promoting *Shaker* K^+^ channel activation (Borjesson and Elinder, 2011; Ottosson et al., 2014).

### Molecular mechanism of PUFAs-dependent modulation of K_Ca_1.1 channels

Contrary to the DHA effects on *Shaker* K^+^ channels, hK_Ca_1.1 channels do not require voltage sensor activation for the PUFA effect. DHA augmented single channel open probability at hyperpolarized potentials where the voltage sensors are predominantly at rest (Hoshi et al., 2013b). In hK_Ca_1.1 channels, opening probability at hyperpolarizing potentials is mainly driven by the opening and closing transitions of the activation gate located near or within the selectivity filter (Seibold et al., 2008). The closure of the activation gate controls the deactivation kinetics and the opening of the gate controls the macroscopic activation kinetics. DHA did not affect the deactivation kinetics of hK_Ca_1.1 channels at hyperpolarized potentials, suggesting that the PUFA accelerates the opening of the activation gate and increases open probability (Hoshi et al., 2013b).

## New targets for PUFAs ion channel modulation

Another crucial aspect needed to determine the beneficial effects of n-3 PUFAs in cardiovascular health is to establish for which pathologies are n-3 PUFAs truly beneficial, neutral or harmful. To do so, it is essential to find new molecular targets responsible for these effects and to analyze how its modulation by PUFAs can affect the physiology in cardiovascular disease models. In this section we summarize recent findings on novel K^+^ channels targets involved in vascular relaxation, blood pressure lowering, and prevention of fatal arrhythmias after acute myocardial infarction (AMI).

### Potassium channels as targets involved in vascular relaxation and blood pressure

#### K_Ca_1.1 channels

In vascular smooth muscle cells (SMC) human K_Ca_1.1 channels or large conductance Ca^2+^-activated K^+^ channels hK_Ca_1.1 channels together with β1 subunits, have been reported to contribute to the regulation of vascular tone. Activation of K_Ca_1.1 channels acts to keep the membrane hyperpolarized, thus generally exerting a negative feedback influence on cellular excitability (Patterson et al., 2002; Nelson and Bonev, 2004). Consumption of oily fish high in DHA has been suggested to decrease blood pressure in some individuals (Ramel et al., 2010; Saravanan et al., 2010; Liu et al., 2011). Application of DHA dilates isolated blood vessels (Wang et al., 2011) potentially by activating K_Ca_1.1 channels (Lai et al., 2009; Wang et al., 2011). To further examine this hypothesis, Hoshi and colleagues generated a K_Ca_1.1 channel deficient mice model mK_Ca_1.1^−/−^, and compared the modulatory effect of DHA in aortic vascular SMCs from wild-type (WT) and mK_Ca_1.1^−/−^mice. Whole-cell outward currents recorded from dissociated aortic vascular SMCs from WT mice were enhanced by extracellular application of DHA but no effect was seen in SMCs from mK_Ca_1.1 deficient mice. Furthermore, the application of DHA into a central vein in WT mice markedly lowered arterial blood pressure. Consistent with the idea that DHA exerts its hypotensive action by activating K_Ca_1.1 channels, injection of DHA into K_Ca_1.1^−/−^ mice had no effect on blood pressure (Hoshi et al., 2013b). Finally, a bolus of DHA ethyl ester had no effect on blood pressure of WT or mK_Ca_1.1 deficient mice. Together, these results confirmed the blood pressure lowering effect of DHA observed in WT mice is directly mediated by the activation of mK_Ca_1.1 channels in vascular SMCs and highlight hK_Ca_1.1as a major target of the reported PUFA hypotensive effects.

#### K_2P_ channels

The two-pore domain potassium channels (K_2P_) traditionally viewed as “background” (voltage-independent) K^+^ channels are dimmers of K_2P_ subunits, each containing four transmembrane segments and two-pore-domain arranged in tandem. K_2P_ channels are mechanosensitive, are active almost instantaneously at all membrane potentials and exhibit strong outward rectification (Lesage and Lazdunski, 2000). K_2P_ channels contribute to the set the resting membrane potential (V_m_) in both non-excitable and excitable cells. In excitable cells, K_2P_ channels can contribute to the repolarization as well (MacKenzie et al., 2015). K_2P_ channels are highly expressed in the lung and cerebral vasculature (Gardener et al., 2004; Blondeau et al., 2007) where they have been suggested to participate in the endothelium-dependent vasorelaxation.

A study by Blondeau et al. (2007) showed that alpha linolenic acid induced vasodilation of the basilar artery in WT but not in K_2P_2.1^−/−^ mice (Blondeau et al., 2007; Garry et al., 2007). More interestingly, it has been suggested that the expression level of K_2P_ channels might be altered in pathological conditions such as pulmonary hypertension (Garry et al., 2007). Pulmonary hypertension is a pathological condition in which there is an increase of blood pressure in pulmonary arteries that can lead hypertrophy of the right ventricle and, finally, heart failure (Hyvelin et al., 2005; Wilkins, 2012). In a comparative study, a 4.4-fold higher density of an endogenous mechanogated K^+^ channel with properties resembling those of K_2P_2.1 was found in aortic endothelial cells from spontaneously hypertensive *vs*. normotensive Wistar-Kyoto rats (Hoyer et al., 1997). In the same line of evidence, it was found that K_2P_6.1^−/−^ knockout mice had a more depolarized V_m_ in aortic SMC, and a higher systemic blood pressure compared to WT mice (Lloyd et al., 2011). Therefore, it was crucial to determine whether K_2P_2.1 and/or other closely related PUFAs-activated K_2P_ channels would play a crucial role in the prevention and or progression of pulmonary hypertension. In a recent study conducted by Nielsen et al. (2013), the expression of different K_2P_ channels was studied under healthy conditions in the murine lung and in a murine model of pulmonary hypertension induced by hypoxia. In the lung of healthy mice, high expression of mRNA of K_2P_2.1 followed by intermediate levels of K_2P_6.1 and low expression of K_2P_4.1, K_2P_10 were found. Voltage clamp experiments showed PUFAs-sensitive K_2P_ channels in endothelium of the main pulmonary artery and of carotid artery. These currents were blocked by unspecific K_2P_ blockers pimozide and flupentixol. Current clamp experiments in pulmonary artery endothelial cells showed that DHA induced a hyperpolarizing shift of the V_m_ of −28 mV. This shift was reversed by pimozide suggesting that DHA caused hyperpolarization through PUFAs-activated K_2P_ channels. DHA activated K_2P_ channels in these cells inducing hyperpolarization and therefore relaxation. Previous studies suggested that vasorelaxation induced by PUFAs in the pulmonary circulation were partially mediated by TEA-sensitive K^+^ channels (Morin et al., 2009). Conversely, the study by Nielsen and colleagues showed no attenuation of DHA-induced vasorelaxation when TEA-sensitive channels K_Ca_3.1, K_Ca_2.3, and K_Ca_1.1 channels were inhibited, suggesting that other mechanisms such as K_2P_ activation, play a role in DHA-dependent vasorelaxation. Endothelial denudation did not prevent the DHA effect, indicating that DHA interacts with K_2P_ channels directly in the SMC layers. However, specific K_2P_ inhibitors are required to confirm this assumption. Altered K_2P_ channel expression patterns were observed in hypertensive animals. K_2P_6.1 channels were significantly up-regulated by four-fold in the lungs of chronic hypoxic mice. The physiological meaning of K_2P_ channel upregulation in pulmonary hypertension is not fully understood. Although further experiments are required to clarify this point, this study reveals K_2P_ channels as potential targets in this condition.

### Potassium channel targets involved in the prevention of fatal arrhythmic events in the early phase of myocardial infarction

#### K_ATP_ channels

Most deaths due to AMI are caused by ischemia-induced ventricular fibrillation (VF) within the early hours after the onset of infarction (O'Doherty et al., 1983). The GISSI-Prevenzione trial on the effects of long term treatment with n-3 PUFAs demonstrated that these substances reduce sudden cardiac death after AMI (GISSI-Prevenzione Investigators, 1999). K_ATP_ channels have been suggested to provide cardioprotection by promoting action potential shortening and decreasing repolarization dispersion (Chi et al., 1990; Venkatesh et al., 1991). In 2011, Tsuburaya and colleagues conducted the first *in vivo* study to test the potential antiarrhythmic effects of long term EPA treatment on ischemia-induced VF in pigs (Tsuburaya et al., 2011). Myocardial infarction was induced in these animals by left coronary artery occlusion and the electrophysiological outcomes on action potentials were analyzed. The survival rate was 50% in the control group *vs*. 100% in the EPA group. In the control group, many VF episodes caused by triggered extra systoles were recorded during ischemia and terminated by current shocks. Conversely, non-sustained ventricular tachycardia episodes (VT) were recorded in the EPA group. The occurrence of VT/VF and the prevalence of pulseless VT/VF was significantly reduced in the EPA group. After the left coronary occlusion, in the control group, MAPD_90_ (monophasic action potential duration measured at 90% of repolarization) was significantly shortened at the ischemic region while in the EPA group this shortening was significantly attenuated. The pretreatment with cromakalim (K_ATP_ channel opener) abolished the inhibitory effects of EPA on ischemia-induced MAPD_90_ shortening, VT/VF occurrence and deteriorated the survival rate during ischemia in the EPA group to 50%. At the molecular level, EPA treatment significantly reduced cardiac mRNA and protein expression and of K_ir_6.2 (a major component of the sarcolemmal K_ATP_ channel) and increased that of the auxiliary SUR2B subunit. Taken together, these findings indicate that EPA suppressed acute phase fatal ventricular arrhythmias (15 min after onset). EPA had an inhibitory effect on ischemia-induced VF *in vivo* probably mediated by amelioration of ischemia-induced monophasic action potentials shortening (Tsuburaya et al., 2011). Nevertheless, patch clamp studies are needed to demonstrate inhibition of K_ATP_ in cardiomyocytes.

## New discoveries in K_V_7 family

The K_v_7.1 family of voltage-gated K+ channels comprises K_v_7.1–K_v_7.5 channels. In the heart, K_V_7.1 channels co-assemble with KCNE1 subunits to give rise to the slowly activating delayed-rectifier K^+^ current *I*_Ks_. This current contributes importantly to cardiac repolarization particularly during β-AR stimulation (Sanguinetti et al., 1996; Volders et al., 2003). The other four components of the K_V_7 family (K_V_7.2–K_V_7.5) are mainly expressed and studied in the nervous system, although they play important roles in other tissues as well (i.e., vascular smooth cells, ear cells, etc.). K_V_7.2 and K_V_7.3 are the major components of the slow voltage-activated M-current. K_V_7.2/K_V_7.3 channels are opened already at rest hence contributing to the V_m_ of dorsal root ganglion (DRG) and hippocampal neurons, and regulating neuronal excitability (Jentsch, 2000; Brown and Passmore, 2009).

### PUFAs and cardiac *I*_Ks_ (K_V_7.1/KCNE1)

As with many other K^+^ channels, K_V_7.1 channels and, therefore, *I*_Ks_ are modulated by PUFAs. Doolan et al. (2002) demonstrated that EPA increases the magnitude of the *I*_Ks_ in *Xenopus* oocytes. Furthermore, long term PUFA treatment was shown to enhance *I*_Ks_ in cardiac myocytes from pigs pigs fed on a PUFA high diet compared to *I*_Ks_ from pigs fed with a normal diet (Verkerk et al., 2006). However, the molecular mechanism underlying the PUFAs-dependent modulation of K_V_7.1/KCNE1 remained to be elucidated. In 2015, two studies addressed this question (Liin et al., 2015; Moreno et al., 2015).

**Figure 4:**
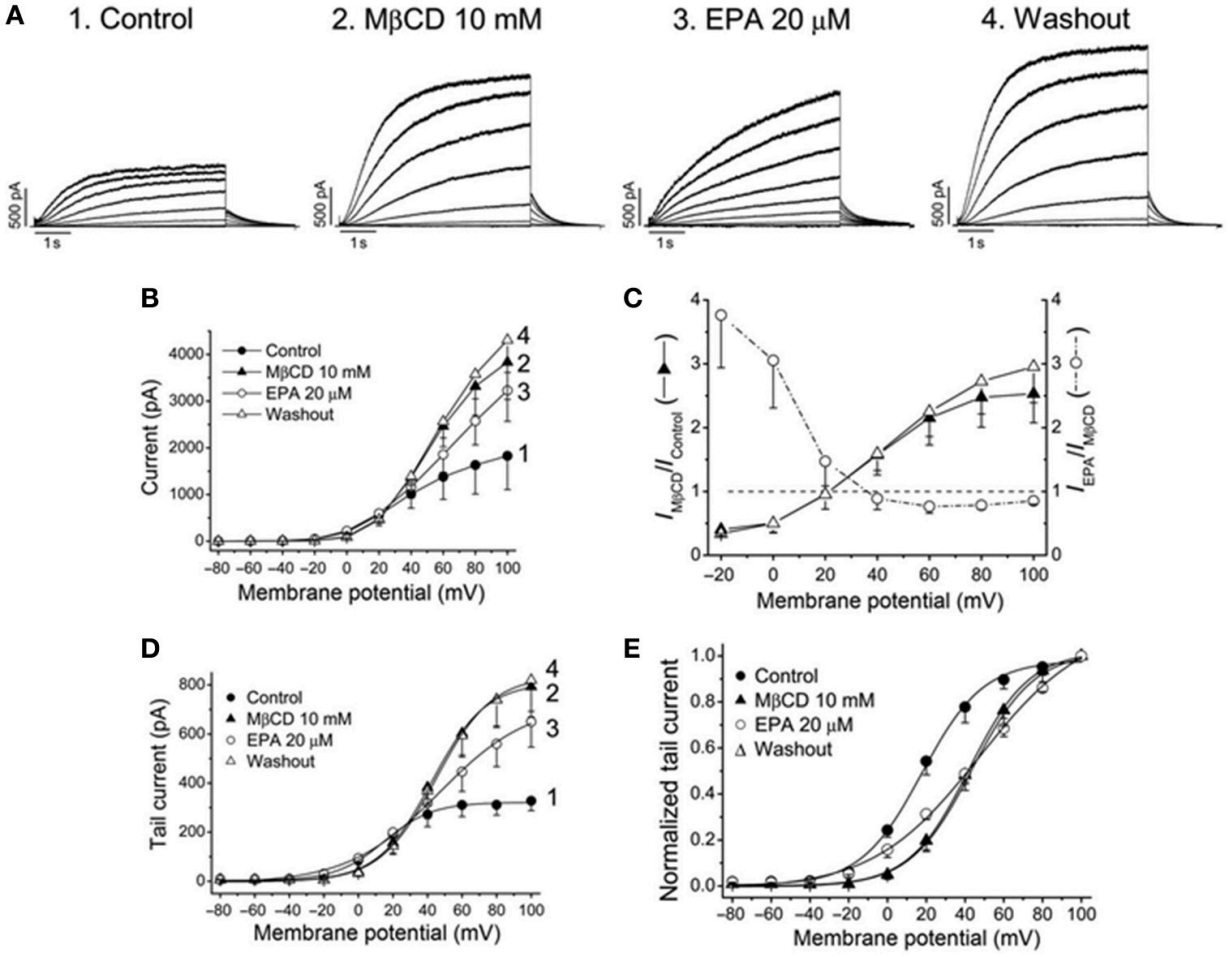
**Effects of EPA in membrane cholesterol-depleted cells with methyl-β-cyclodextrin (MβCD). (A)** Current records obtained from the same cell and generated by K_V_7.1-KCNE1 channels in control, after perfusion with MβCD, with EPA and after washout the cells with EPA-free external solution. **(B)** Current-voltage relationships under all conditions shown in **(A)**. **(C)** Plot showing the voltage dependence of the MβCD and EPA effects **(D)** Activation curves and **(E)** normalized activation curves. Moreno et al. (2015).

Moreno et al. (2015) analyzed the effects EPA and DHA in K_V_7.1/KCNE1 channels expressed on a heterologous expression system and in *I*_Ks_ in guinea pig myocytes. Two aspects of PUFA-dependent modulation of ion channels merit particular attention. (i) Dietary PUFAs incorporate to the cell membrane. Therefore, PUFAs-dependent modulation of ion channel depend on the way of administration: acute *vs*. chronic [see Moreno et al. (2012) for extensive review]. In fact, Moreno and colleagues reported that acute but not chronic administration of PUFAs increased the magnitude of K_V_7.1/KCNE1 channels and shifted the activation curve toward opposite directions (leftward and rightward shift for acute and chronic administration, respectively). In addition, long-term administration of n-3 PUFAs reduced K_V_7.1 but not KCNE1 expression due to increased degradation via proteasome. The significant decrease in K_V_7.1 expression level might account for the lack of current density increase when n-3 PUFAs were applied chronically (Moreno et al., 2015). (ii) Direct *vs*. indirect effects. As previously mentioned, there were two trends of opinion to explain the mechanism of action by which PUFAs modify ion channels: direct interaction *vs*. alteration of the cell membrane fluidity. Evidences in favor of both type of modulation were found simultaneously in the study by Moreno et al. (2015). Chronic administration of EPA and DHA disrupted lipid rafts domains where K_V_7.1 channels are normally found (Balijepalli et al., 2007) delocalizing K_V_7.1 in the cell membrane. Lipid raft disruption can be mimicked within minutes by the acute application of methyl-β-cyclodextrin (MBCD). The application of MBCD increased the current magnitude of K_V_7.1/KCNE1 at potentials positive to +30 mV and shifted the activation curve toward positive potentials emphasizing the impact of the membrane environment on channel properties (indirect effect; Figure 4; Moreno et al., 2015). On the other hand, after lipid raft disruption with MBCD, acute application of EPA produced similar effects than those observed in non-cholesterol-depleted cells: Current density increase and shift of the midpoint in the hyperpolarizing direction. This fast modulation was explained by a direct effect on the ion channel of EPA during acute application. Together these data provided evidence for direct and indirect PUFA dependent modulation of K^+^ channels suggesting that both mechanisms are not mutually exclusive and can happen simultaneously (Moreno et al., 2015).

The study by Liin et al. (2015) examines the mechanism by which n-3 PUFAs promote K_V_7.1 channel opening and how KCNE1 modulated K_V_7.1 sensitivity to PUFAs. In addition, they explored the possibility to develop PUFAs analogs as drugs to prevent cardiac arrhythmias. Similarly to what was described for *Shaker* K^+^ channels, DHA promotes channel opening by acting electrostatically on the VSD of K_V_7.1 channels. KCNE1 tunes K_V_7.1 sensitivity to PUFAs by lowering the local pH in the vicinity of the PUFA head group which results in DHA protonation. In other words, the PUFA effect is KCNE1-independent as long as the PUFA is negatively charged (Liin et al., 2015). This finding raised the possibility to study the antiarrhythmic potential of n-3 PUFAs analogs as novel therapeutic agents. In this regard, Liin and colleagues tested the effects of two PUFAs analogs in two arrhythmia models: isolated embryonic rat cardiomyocytes and the intact heart of guinea pig. In isolated embryonic rat cardiomyocytes (in which *I*_Ks_ but not *I*_Kr_ rapidly activating delayed rectifier potassium current is expressed) arrhythmia was induced by applying subsaturating concentrations of chromanol 293B and the effects of N-arachidonoyl taurine were tested. Chromanol 293B increased action potential duration and led to arrhythmic firing. The permanently negatively charged N-arachidonoyl-taurine reversed the effect of chromanol 293B decreasing the action potential duration (APD) and abolishing the arrhythmic firing. The effects of docosahexaenoyl glycine were tested on the intact heart of guinea pig (DHA-Gly has a lower pKa than DHA and therefore is negatively charged at physiological pH even in the presence of KCNE1). To mimic the long QT (syndrome characterized by a long interval between the Q and the T waves of the ECG) setting in this model (APD duration and QT prolongation), the E4031 *I*_Kr_ blocker was applied. Perfusion of the guinea pig heart with 10 μM DHA-Gly reversed the effects of E4031 restoring QT interval duration (Liin et al., 2015) demonstrating their antiarrhythmic potential.

Concerning the PUFAs antiarrhythmic mechanism, minor discrepancies can be found through the literature. Overall, *in vitro* and *in vivo* experiments and clinical trials support an antiarrhythmic effect of PUFAs. Liin and colleagues claim that the antiarrhythmic effects of DHA cannot be due to effects on *I*_Ks_ gating because at physiological pH 7.4, KCNE promotes DHA protonation making DHA ineffective (Liin et al., 2015). Instead they propose that the antiarrhythmic effect of PUFAs in the heart is due to the inhibition of *I*_Na_ or *I*_CaL_ (Leaf et al., 2003, 2005; Danthi et al., 2005; Boland and Drzewiecki, 2008). However, we found that both EPA and DHA increase *I*_Ks_ in guinea pig cardiomyocytes and shifted the G(V) curve toward the hyperpolarizing direction of *I*_Ks_ expressed in COS7 cells. Furthermore, action potential simulations showed that the increase of *I*_Ks_ induced by acute administration of PUFAs could play a relevant role in action potential shortening when other repolarizing current for instance *I*_Kr_, are compromised. On the other hand, chronic exposure to PUFAs does not affect APD so that the protective role seems lost in this condition.

### PUFAs and neuronal M-current

It has been reported that PUFAs increase both seizure and pain thresholds (Voskuyl et al., 1998; Xiao and Li, 1999; Taha et al., 2010; Bandero et al., 2013) in rats and mice, and reduce neuronal excitability *in vitro* (Xiao and Li, 1999; Young et al., 2000). As it was shown for the heart, the modulation of neuronal excitability by PUFAs is mediated by effects on ion channels crucial for neuronal excitability (Boland and Drzewiecki, 2008). PUFAs have been suggested to increase the M-current but their effects are complex and not completely understood. In a recent paper, it was shown again that only negatively charged n-3 PUFAs such as DHA, EPA, ALA, can quickly shift the G(V) curve of K_V_7.2/K_V_7.3 channels expressed on *Xenopus* oocytes toward more negative voltages facilitating channel opening (Liin et al., 2016; Valenzuela, 2016). More importantly, the anti-excitable effects of PUFAs on the neuronal M-current were studied in dorsal root ganglions from mice. Addition of DHA to dorsal root ganglion neurons hyperpolarized the V_m_ (by −2.4 mV) increasing the threshold current required to evoke action potentials. Since these effects were reverted by the M-current blocker XE991, the reduced neuronal excitability was attributed to the PUFA mediated augmentation of M-current (Liin et al., 2016). In the same line of evidence, computer simulations predict that equivalent changes around 1–5 mV in the V_m_ of neurons and in the voltage-dependence of ion channel activation is sufficient to decrease neuronal excitability and prevent epileptic episodes (Tigerholm et al., 2012). In summary, all these results suggest that PUFAs can be used as a blueprint for rational drug design of new M-currents activators more selective and efficient for the treatment of neurological disorders such as epilepsy and pain.

## Concluding remarks

From 1990's we know that PUFAs produce diverse effects on ion channels that might be beneficial for health. The mechanism of action initially proposed by the Elinder's group: The lipoelectric hypothesis (the lipid bilayer or bilayer/channel interface close to the S3–S4 segment) from where they act electrostatically on the voltage sensor and thereby change the voltage dependence of the channel. The modulation of potassium channels by PUFAs is channel specific, as indicated by the identification of two PUFAs binding sites in different potassium channels families. Besides the gating modulation of ion channels different studies have demonstrated that PUFAs can modulate differentially ion channels for instance through the modulation of the biophysics of the lipid bilayer, by targeting the channels to different areas of the cell membrane (i.e., lipid rafts) and even modifying the gene expression. Finally, other ion channels such as K_Ca_1.1, K_ATP_, and K_2P_ have been recently reported as sensitive to the effects of PUFAs. Since these potassium channels are present in the cardiovascular and in the nervous systems, PUFAs might play an important role in the control of blood pressure, cardiac arrhythmias, and neurological disorders.

## Author contributions

CM, AC, and CV wrote this review.

## Funding

This work was supported by SAF2013-45800-R, SAF2016-75021-R, FIS RIC-RD12/0042/0019, and FIS CIBER CB/11/00222 Grants. RIC and CIBER are funded by the Instituto de Salud Carlos III. The cost of this publication was paid in part by funds from the European Fund for Economic and Regional Development. AdlC holds a CSIC contract.

### Conflict of interest statement

The authors declare that the research was conducted in the absence of any commercial or financial relationships that could be construed as a potential conflict of interest.
